# Artificial Intelligence in Sepsis Management: An Overview for Clinicians

**DOI:** 10.3390/jcm14010286

**Published:** 2025-01-06

**Authors:** Elena Giovanna Bignami, Michele Berdini, Matteo Panizzi, Tania Domenichetti, Francesca Bezzi, Simone Allai, Tania Damiano, Valentina Bellini

**Affiliations:** Anesthesiology, Critical Care and Pain Medicine Division, Department of Medicine and Surgery, University of Parma, Viale Gramsci 14, 43126 Parma, Italy; michele.berdini@unipr.it (M.B.); matteo.panizzi@unipr.it (M.P.); tania.domenichetti@unipr.it (T.D.); francesca.bezzi@unipr.it (F.B.); simone.allai@unipr.it (S.A.); tania.damiano@unipr.it (T.D.); bellini.vnt@gmail.com (V.B.)

**Keywords:** sepsis management, artificial intelligence, machine learning, early warning systems, clinical application

## Abstract

Sepsis is one of the leading causes of mortality in hospital settings, and early diagnosis is a crucial challenge to improve clinical outcomes. Artificial intelligence (AI) is emerging as a valuable resource to address this challenge, with numerous investigations exploring its application to predict and diagnose sepsis early, as well as personalizing its treatment. Machine learning (ML) models are able to use clinical data collected from hospital Electronic Health Records or continuous monitoring to predict patients at risk of sepsis hours before the onset of symptoms. **Background/Objectives**: Over the past few decades, ML and other AI tools have been explored extensively in sepsis, with models developed for the early detection, diagnosis, prognosis, and even real-time management of treatment strategies. **Methods**: This review was conducted according to the SPIDER (Sample, Phenomenon of Interest, Design, Evaluation, Research Type) framework to define the study methodology. A critical overview of each paper was conducted by three different reviewers, selecting those that provided original and comprehensive data relevant to the specific topic of the review and contributed significantly to the conceptual or practical framework discussed, without dwelling on technical aspects of the models used. **Results**: A total of 194 articles were found; 28 were selected. Articles were categorized and analyzed based on their focus—early prediction, diagnosis, mortality or improvement in the treatment of sepsis. The scientific literature presents mixed outcomes; while some studies demonstrate improvements in mortality rates and clinical management, others highlight challenges, such as a high incidence of false positives and the lack of external validation. This review is designed for clinicians and healthcare professionals, and aims to provide an overview of the application of AI in sepsis management, reviewing the main studies and methodologies used to assess its effectiveness, limitations, and future potential.

## 1. Introduction

**Figure 1:**
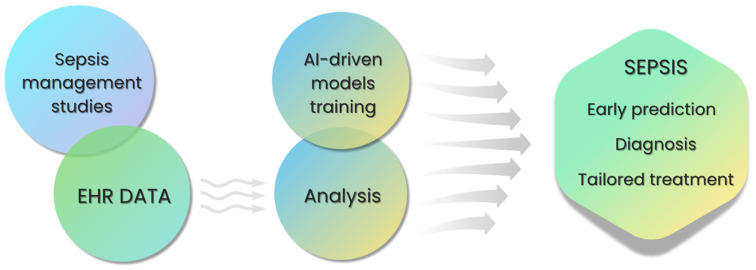
AI workflow applied to sepsis management.

Sepsis is a life-threatening condition caused by the body’s dysregulated immune response to infection, which may lead to organ dysfunction, septic shock, and death. As a medical emergency, sepsis requires rapid diagnosis and timely intervention to prevent severe complications and reduce mortality [1]. It remains a significant cause of morbidity and mortality globally, and in recent decades, predicting which patients are at risk of sepsis has become a research priority. A promising field involves using artificial intelligence (AI)-driven models. AI, broadly defined, is a branch of computer science focused on developing systems that can perform tasks requiring human-like intelligence, such as learning, reasoning, and decision-making [2,3]. Machine learning (ML) is a subset of AI, capable of “learning” from data and acquiring new knowledge and techniques across machine learning models (MLMs). In healthcare, AI enables the analysis of large, complex datasets, allowing us to recognize intricate patterns that may be missed by traditional methods [4]. This capability is especially valuable in sepsis management, where early prediction, diagnosis, and personalized treatment can significantly impact patient outcomes [5,6,7], as shown in Figure 1.

This review is designed for clinicians and healthcare professionals and aims to provide an overview of the application of AI in sepsis management, reviewing the main studies and methodologies used to assess its effectiveness, limitations, and future potential. In the end, we will also discuss some commercially available models.

## 2. Materials and Methods

This review was conducted according to the SPIDER (Sample, Phenomenon of Interest, Design, Evaluation, Research Type) framework methodology. A large-scale search was performed across PubMed, MeSH, Medline, Embase and Scopus using keywords “sepsis” and “Artificial Intelligence”, or “sepsis” and “machine learning”, in a timeframe ranging from 1 January 2019 to 10 December 2024, in order to obtain an updated overview of the current state of the art of the topic focusing on prediction, diagnosis and treatment. The queries chosen by the authors were: “(((“sepsis” [All Fields]) AND (“Artificial Intelligence” [All Fields])) AND ((prediction) OR (diagnosis) OR (treatment)))”, “Full text, Clinical Study, Clinical Trial, Multicenter Study, Observational Study, Pragmatic Clinical Trial, Randomized Controlled Trial, from 2019–2024”; (sepsis) AND (machine learning), “Full text, Clinical Study, Clinical Trial, Multicenter Study, Observational Study, Pragmatic Clinical Trial, Randomized Controlled Trial, from 2019–2024”; (sepsis) AND (Artificial Intelligence), and “Full text, Clinical Study, Clinical Trial, Multicenter Study, Observational Study, Pragmatic Clinical Trial, Randomized Controlled Trial, from 2019–2024”.

A total of 194 articles were found. We included papers that discussed the use of AI in sepsis management regardless of setting or study population. We excluded non-original articles, reviews and papers with no full text available. We focused on those articles that provide clinical insights and application, even without dwelling on the technical aspects of the models used. Some papers were found to be related articles.

A critical overview of each paper was conducted by 3 different reviewers, selecting those that provided original and comprehensive data relevant to the specific topic of the review and contributed significantly to the conceptual or practical framework discussed. For each study design, ML models and methods, analysis, intent, selling points and limitations were explored. Subsequently, the articles were categorized and analyzed based on their focus: early prediction, diagnosis, mortality or improvement in the treatment of sepsis.

At the end, a total of 28 papers were selected. The results are summarized in Table 1.

### 2.1. Early Prediction of Sepsis

One of the most promising applications of AI in the management of sepsis is the ability to predict the likelihood of onset of the syndrome hours in advance of obvious clinical symptoms. MLMs can in fact analyze clinical data extracted from electronic health records (EHR) or continuous monitoring, such as vital signs, laboratory parameters and medical history of patients, to identify those at risk of developing sepsis. In order to demonstrate their performance, MLMs are often compared to traditional sepsis scoring systems, like Systemic Inflammatory Response System (SIRS) criteria, Sequential Organ Failure Score (SOFA), quick SOFA (qSOFA), National Early Warning Score (NEWS) and Modified Early Warning Score (MEWS).

In a study from 2019, Giannini et al. [8] developed a random forest-based ML algorithm for the prediction of severe sepsis and septic shock. The model was trained on a dataset of more than 160,000 patients, including 587 clinical variables, including vital signs and laboratory data, to make predictions on patients not admitted to the Intensive Care Unit (ICU). The results were encouraging, with a specificity of 98%, but sensitivity was relatively low, at 26%. This indicates that although the algorithm was able to avoid false positives, it had difficulty detecting all patients at real risk. In particular, the algorithm demonstrated greater accuracy in predicting cases of septic shock than less severe sepsis, reflecting a specific potential for identifying severe cases. Despite the specificity of the algorithm, its clinical impact was limited. During an eight-month test period in two hospitals, both in “silent” mode and with active alerting, only a modest increase in the use of lactate tests and fluid administration was observed, but no significant improvements in mortality, hospital length of stay or ICU transfers were recorded. This emphasizes that although AI can detect sepsis early, translating these predictions into tangible clinical improvements requires further research and optimization.

Another ML algorithm [9] was developed to predict sepsis up to 48 h before onset, using six vital signs (heart rate, respiratory rate, temperature, systolic and diastolic blood pressure, oxygen saturation). Based on retrospective data from two different datasets, the algorithm was compared with traditional scoring systems such as SIRS, SOFA, MEWS and qSOFA. The algorithm showed a higher Area Under the Curve (AUROC) (0.88 at onset, 0.84 and 0.83 at 24 and 48 h before, respectively) than traditional scores (SOFA, 0.72; SIRS, 0.66). The system stands out for its ability to identify patients at risk of sepsis early on without requiring complex data, using only variables frequently measured in hospital settings. In addition, the model also demonstrated robustness in cross-population validation between the two datasets. This advanced prediction offers opportunities for intensive monitoring and early intervention, reducing the risk of progression to septic shock. No less importantly, the exclusive use of essential data makes the algorithm suitable for diverse settings, including non-ICU environments.

An article from 2020 [10] presents a study on the early prediction of sepsis using an Explained Artificial Intelligence Model (EASP). The study uses EHR data from ICU patients, collected during the PhysioNet/Computing in Cardiology Challenge 2019. The main objective was to develop a model that can predict sepsis in real time and explain the impacts of the variables used in the prediction. EASP analyzes 168 features extracted on an hourly basis, including raw data, time series, derived variables and empirical clinical indicators. The model uses XGBoost with Bayesian optimization. The model showed promising performance, with an area under the curve (AUC) of 0.85 and a sensitivity of 90%, although specificity was lower (64%). Despite its overall good performance, the results showed limitations, including the generation of false positives and inconsistent performance across test sets from different hospitals.

Another study that focuses on using EHR data to develop a deep learning model for early sepsis detection is the one by Lauritsen et al. [11]. Unlike traditional models limited to specific clinical parameters or ICU settings, this model incorporates a wider range of data, including raw sequential EHR events from multiple hospital departments. The proposed model employs a combination of convolutional neural networks (CNNs) and Long Short-Term Memory (LSTM) networks designed to extract and learn temporal and sequential patterns from patient data without requiring intensive feature engineering. The model predicts the onset of sepsis up to 24 h earlier, obtaining an AUROC of 0.856 at 3 h before onset and 0.752 at 24 h. Clinical utility was evaluated by analyzing opportunities for intervention, such as administering antibiotics or performing blood cultures. The results reveal that the model can identify many cases of sepsis in which previous interventions have not been initiated, facilitating earlier treatment and potentially reducing morbidity and mortality.

A further relevant study is the one by Shashikumar et al. [12], who proposed DeepAISE (Deep Artificial Intelligence Sepsis Expert), an advanced AI model for predicting the onset of sepsis at one-hour intervals, up to 12 h before the clinical onset of symptoms, using a recurrent neural network combined with a parametric Weibull–Cox risk model in terms of survival. This system, designed for use in an ICU, analyzes 65 updated clinical variables on an hourly basis, including vital signs and laboratory parameters, continuously extracted from electronic medical records. It demonstrated high accuracy, achieving an AUC of 0.90 per prediction at 4 h and maintaining competitive performance up to 12 h at the Lowest False Alarm rates (FARs) between 0.20 and 0.25. A distinctive aspect of DeepAISE is its interpretability; the model not only predicts sepsis risk, but also identifies key risk factors in real time for each patient, making the predictions more usable by clinicians than traditional ‘black box’ MLMs. In addition, the validation of the model was conducted on external cohorts, thus demonstrating high generalizability across geographically and clinically diverse populations. The article acknowledges some limitations and potential biases related to the implementation of DeepAISE; the case–control approach often overestimates the prevalence of sepsis compared to a sequential prediction design. This can lead to high false alarm rates when used in real clinical settings. Also, the framework is affected by missing data in clinical sets, which may reduce the sensitivity of the model in less frequent monitoring settings than in ICUs.

In a multicenter prospective study [13], an ML algorithm using gradient-boosted trees was applied to predict severe sepsis in real-world hospital settings across the United States. The algorithm analyzed vital signs and demographic data from 17,758 patients who met systemic inflammatory response syndrome (SIRS) criteria. Its implementation led to a significant reduction in in-hospital mortality (39.5%), hospital length of stay (32.3%), and 30-day readmissions (22.7%). The study demonstrated the efficacy of a data-minimal approach, with the algorithm relying on routinely available EHR data. The MLA outperformed traditional sepsis scoring systems (e.g., SIRS, SOFA), which are limited by poor sensitivity and reliance on non-rapidly available laboratory data. It is worth noticing that while other ML approaches have largely been evaluated retrospectively, this study provides robust prospective evidence of clinical benefits. The reduction in sepsis-related outcomes suggests the algorithm can enhance early detection and decision-making, which are crucial for timely antibiotic administration and fluid management.

Persson et al. [14] constructed a high-performance MLM for sepsis prediction called NAVOY Sepsis by using convolutional neural networks. The used data came from the EHRs of ICU patients of a single center, with a total of 20 variables aiming to predict sepsis according to sepsis-3 criteria.

The objective of the authors is to develop a robust and widely applicable sepsis prediction model for European ICUs, based on routinely collected clinical data.

A key strength of the study is its external validation of performance using hold-out test data. The results were highly promising, with an AUROC of 0.90 and the ability to predict sepsis up to 3 h before onset. The model outperformed existing sepsis early warning scoring systems. However, limitations persist, similarly to other studies, such as the lack of prospective randomized trials, the inability to exclude biases in the data (such as variations in length of stay distribution), insufficient broad external validation (despite the use of hold-out test data in this study), and the absence of information on the clinical or economic impact of the model in real-world practice.

A study conducted by Pappada et al. [15] developed a Sepsis Risk Index (SRI) for the early identification of sepsis in patients admitted to intensive care units. Using a model based on artificial neural networks (ANN), the SRI combines real-time clinical data extracted from EHRs with the prediction of changes in vital parameters. The model showed a sensitivity of 79.1% and a specificity of 73.3% for the diagnosis of sepsis, with an area under the ROC curve of 0.82. For the diagnosis of septic shock, sensitivity increased to 83.8% and specificity remained unchanged.

A very recent study [16] describes the development and validation of the Sepsis ImmunoScore, the first FDA-cleared artificial intelligence algorithm for sepsis prediction. The model was trained on data from 2366 hospitalized patients and validated on two cohorts, internal (393 patients) and external (698 patients), collected from five US hospitals between 2017 and 2022. The primary objective was to predict sepsis within 24 h of hospital admission, using Sepsis-3 criteria. The model uses 22 clinical parameters, including vital signs, laboratory tests and specific biomarkers such as procalcitonin (PCT) and C-reactive protein (CRP). With a calibrated random forest algorithm and SHAP values for interpretability, the system achieved an AUROC of 0.85 in the derivation set, 0.80 in the internal validation and 0.81 in the external validation. In addition, patients were stratified into four risk categories, demonstrating significant correlations with sepsis severity and clinical outcomes.

### 2.2. Diagnosis of Sepsis

The main difference between the early prediction and diagnosis of sepsis lies in the actual occurrence of sepsis in the patient being examined. Early prediction assesses the risk of developing sepsis before clinical signs appear, though it is not definitive, with the goal of adjusting medical attention and procedures accordingly. In contrast, diagnostic MLMs use real-time clinical data to calculate scores, which can then be compared with traditional scoring systems, such as Systemic Inflammatory Response System (SIRS) criteria, Sequential Organ Failure Score (SOFA), quick SOFA (qSOFA), National Early Warning Score (NEWS) and Modified Early Warning Score (MEWS), suggesting potential advantages in certain contexts.

In a paper evaluating the role of AI in sepsis management [17], an ML algorithm was developed using electronic medical record (EMR) data and biomarkers (procalcitonin, interleukin-6 and C-reactive protein) to predict and stratify sepsis cases. The study included 1400 patients from two hospitals, using a random forest model trained on EMR parameters and non-routinely measured biomarkers. This approach achieved an AUROC of 0.83 for the diagnosis of sepsis, demonstrating reliable diagnostic and prognostic performance. The model produces a sepsis risk score that correlates with disease severity, predicting outcomes such as length of hospital stay, 30-day mortality and hospital readmission rates. Patients stratified into low-, medium- and high-risk categories showed significant differences in these outcomes. In particular, patients with higher predicted scores had a median hospital stay of 8.5 days versus the 3.2 days of low-risk patients and a 30-day mortality rate of 13.3% vs. the 2.9% of the low-risk population, and this correlated with worse outcomes, such as septic shock.

This algorithm emphasizes how combining biomarkers with EMR features significantly outperformed the use of EMR data alone, enhancing both diagnostic and prognostic accuracy.

The article by Wang et al. [18] describes a supervised random forest MLM applied to the early diagnosis of sepsis in ICU patients. The study used a dataset of 4449 patients with infections, trained the data set by pre-procedure clinical variables, used a cross-validation to evaluate the prediction accuracy of model, and, finally, tested it with the validation data set with a 4:1 split for training and validation. Key variables used in the model included neutrophils, D-dimer, eosinophils, albumin, and white blood cells. The model showed a good discriminative power, with an AUROC of 0.91, a sensitivity of 87% and a specificity of 89%. The study’s key advantage is its ability to diagnose sepsis after an infection has already been confirmed. However, the authors emphasize the need for further external validation to ensure its performance beyond the Chinese population. Additionally, they suggest the inclusion of more variables for enhanced accuracy.

In this scenario, another study from Arrigue and Urrechaga [19] tested different MLMs to diagnose sepsis in patients admitted to the ED with suspected infection, using data from blood tests. Logistic Regression (LR) was used as a benchmark with MLMs developed—naïve Bayes (NB), K-nearest neighbor (KNN), support vector machines (SVM), random forest (RF), multi-layer perceptron (MLP) and extreme gradient boosting machine (XGBOOST). MLMs used leukocytes Cell Population Data (CPD), white blood cells ratio, positive cultures for bacteria, and serology or molecular tests for viruses. Sepsis was diagnosed using a qSOFA score ≥2. MLP achieved the best results compared to other models, in terms of performance, precision and calibration. The greatest strength of this study lies in its prospective design and the use of clinical data collected promptly after assessment, which incurs low costs and accelerates the decision-making process. This aspect warrants further exploration, such as by comparing the model with other sepsis risk and mortality stratification scores.

One of the most recent examples of a sepsis prediction tool is SepsisFinder, a casual probabilistic network (CPN) model presented by Valik et al. [20], which mimics the reasoning of healthcare providers by using routine data coming from EHRs of non-ICU patients to diagnose sepsis basing on Sepsis-3 criteria. SepsisFinder was compared with NEWS2, and it was shown to obtain an earlier trigger for the identification of sepsis within 48 h, with a higher AUROC of 0.950, at the cost of slightly lower precision. The model was also compared through a gradient-boosting decision tree (GBDT) model with other MLMs, but none of them performed better. The accuracy of the model is linked to earlier admission periods and bloodstream infections, which led to a well-timed initiation of antibiotic treatment. However, the experiment faces the same limitations as were previously mentioned: a lack of external validation, limited generalizability, and the potential impact of missing or inaccurate data in the EHRs.

A further article [21] evaluates the effectiveness of a machine learning-based sepsis alert system (MLASA) in an emergency department (ED) by means of a cluster-randomized study. The system analyzes real-time data from EHR to identify sepsis early. Patients in intervention groups received an MLASA alert displayed in real time, while those in control groups were monitored by conventional methods (e.g., qSOFA or MEWS scores). The implementation of the system increased the proportion of patients who received antibiotics within 1 h (68.4% compared to 60.1% in the control group). Administration within 3 h was also more frequent in the intervention group (94.5% versus 89.0%). However, the median triage–antibiotic times (46 vs. 50 min) showed no significant differences. Therefore, ML outperformed conventional methods in the diagnosis of sepsis, achieving an area under the ROC curve of 0.93 compared to qSOFA (0.73), SIRS (0.84) and MEWS (0.86). This study shows that MLASA improves the timely diagnosis and treatment of sepsis in critical care settings, although the effect on overall response time remains marginal.

In a more specific field, Zheng et al. [22] combined ML and specific biosignatures, like biomarkers and metabolomic characteristics, to develop an accurate diagnosis strategy among septic patients. XGBoost combined with three feature selection methods, variance threshold, maximal information coefficient (MIC), and relief, was used to select clinical and metabolomic features that distinguished septic patients from controls. The model successfully detected 57 features with high accuracy (AUC = 0.94). White blood cell (WBC) count, platelet count, and blood lactate were shown as key clinical features. The model also scored an AUC of 0.80 on discrimination between Gram-positive and Gram-negative infections (sensitivity, 86%; specificity, 48%). Top biomarkers included metabolites associated with ubiquinone and D-arginine metabolism. Sepsis was associated with disrupted nitrogen metabolism, mitochondrial dysfunction, and organ failure (e.g., liver, intestine, kidney), and specific metabolic biomarkers (e.g., Coenzyme Q10, bile acids, glycerophospholipids) highlighted key differences in host–pathogen interactions. ML demonstrated strong potential for identifying pathogen-specific biomarkers, offering diagnostic capabilities that surpass traditional methods and revealed systemic physiological disruptions (e.g., nitrogen metabolism, mitochondrial damage) critical to sepsis progression and outcomes.

### 2.3. Sepsis Mortality Prediction

Predicting sepsis-related mortality is closely connected with sepsis’ prediction itself, and so it is crucial for improving clinical outcomes. Recent advances underscore AI’s potential to support early, personalized interventions in critical conditions, with evidence suggesting that timely, data-driven models can improve patient outcomes significantly [23]. AI and MLMs, thanks to their ability to analyze large amounts of information in real time, allow high-risk patients to be identified, enabling early and targeted interventions to significantly reduce sepsis-related mortality.

Park et al. [24] conducted a comprehensive comparison between traditional logistic regression and commonly used MLMs for predicting sepsis mortality, based on retrospective data of hospitalized sepsis patients. They developed four MLMs to predict in-hospital mortality: logistic regression with Least Absolute Shrinkage and Selection Operator (LASSO) regularization, random forest (RF), gradient-boosted decision trees (XGBoost), and a deep neural network (DNN). To evaluate model performance, they also used the Super Learner (SL) model, which estimates and combines predictions from multiple models. The results were significant; all four MLMs outperformed the reference logistic regression model, though room for improvement remains. This opens possibilities for risk stratification and proactive clinical interventions. The models also identified certain clinical variables associated with higher mortality. The authors acknowledged some study limitations but felt these had minimal impact on the results due to the large sample size and robust standardization. A potential bias noted by the authors was the variation in results across hospitals and regions due to differences in sepsis care and outcomes.

In accordance with the paper above, Rodriguez et al. [25] presented and compared the applicability and the performances of four different data mining MLMs for the classification and prediction of mortality in adult patients hospitalized for sepsis. In the first subset, the model with the best performance was random forest, with an accuracy of 84% and an AUC-ROC of 0.61. All models showed high survival prediction (89–92%) but poor mortality prediction accuracy (28–30%). For the second subset, a better prediction of death was observed mainly in the support vector machine (SVM) with ANOVA kernel and Artificial Neural Network (ANN); both achieved an accuracy of about 70.7% and an AUC-ROC = 0.69. Physiological variables like lactate, MAP (mean arterial pressure), and Glasgow Coma Scale showed better predictive power than treatment-related variables. When predicting sepsis mortality, models driven by physiological variables performed better than those using clinical care variables. Overall, SVM and ANN demonstrated better predictive potential due to their ability to learn complex, nonlinear relationships.

Sepsis mortality prediction is a particular concern, especially in ICUs. In an observational study [26], a comparison of five different MLMs was conducted to show which one performed the best. Gradient boosting decision tree (GBDT), logistic regression (LR), k-nearest neighbor (KNN), random forest (RF), and support vector machine (SVM) were involved in a population of ICU septic patients. In the final results, GBDT outperformed all other models, with the highest performance metrics (AUROC 0.992, F1 Score 0.933), and it ranked the most critical predictors of mortality, including age, Glascow Coma Scale, blood urea nitrogen, lactate, heart rate and blood pressure. GBDT was able to provide excellent discrimination ability and the best predictive accuracy due to its ability to handle nonlinear interactions and missing values effectively, and it showed resilience to noise and outliers in the data. On top of this, the model identified key clinical indicators actionable in clinical practice (e.g., lactate levels, GCS, and BUN), revealing itself as a real-time clinical decision support system for ICU teams, enabling personalized risk stratification for septic patients and timely interventions, thus improving survival rates.

In efforts to improve sepsis outcomes, Adams et al. [27] found that their early warning system, TREWS (Targeted Real-time Early Warning System), reduced the median time to the first antibiotic order by 1.85 h. After adjusting for patient severity, TREWS usage showed improved mortality rates, particularly for high-risk patients flagged by the alert. Reduced mortality was associated with better SOFA score progression and shorter hospital stays. The study mitigated potential surveillance bias by including only post-deployment data, though the authors noted limitations such as restricted markers for alerting, a lack of randomization, the retrospective identification of sepsis, and insufficient data on antibiotic administration.

Some results [28] suggest that ML models can outperform traditional methods such as the SOFA score, in terms of both predictive accuracy and interpretability. One example is a study developing MLMs to predict mortality of patients with sepsis in emergency rooms, using data collected from a large multicenter cohort (19 hospitals) between 2019 and 2020. Six models (including XGBoost, CatBoost and LightGBM) were built to analyze 44 clinical variables, including vital signs, laboratory tests and SOFA score components. The results show that models based on general clinical variables outperformed those based solely on the SOFA score, with CatBoost performing best (AUC 0.800). LightGBM and XGBoost showed similar strong performances (AUC 0.795 and 0.797, respectively). The algorithm used Shapley Additive Explanations (SHAP) to explain the contribution of individual variables to prediction, showing that markers such as lactate, blood urea nitrogen and albumin were the most influential. These key findings align with prior research on the relevance of those variables in sepsis prognosis.

Jiang et al. [29] focused on analyzing sepsis and sepsis death risk factors in ICUs by using different MLMs on a patient dataset (MIMIC-IV). LR with Python sklearn and GBDT boosted with the XGBoost package were trained and tested to solve clustered real-time sepsis prediction and septic mortality prediction. The contribution of every feature was analyzed by SHAP. The models achieved an AUROC of 0.745 on sepsis prediction (95% CI: 0.731–0.759) and an AUROC of 0.8 on sepsis death prediction (95% CI: 0.770–0.828). The major contributing risk factors, such as maximum pO_2_ and temperature, in predicting sepsis and the mean DBP and MBP in predicting septic death made relative risk contributions to the outcome. The results suggest that oxygen exchange parameters (e.g., pO₂, SpO₂, AaDO₂) were the major contributors to sepsis prediction, while circulatory variables and anion gap contributed to septic death prediction. These factors were unidirectionally correlated with risk alterations. SHAP analysis revealed that impaired temperature and anion gap increased risk for sepsis shortly before diagnosis, while fluctuations in diastolic blood pressure and mean blood pressure, and significant increases in the anion gap, were observed for septic death. Among patients with septic death, two distinct groups (A and B) were identified, whereas group A displayed a higher contribution from blood urea nitrogen and anion gap, while group B showed a stronger association with circulatory factors.

The use of this interpretable ML provided real-time risk-monitoring information related to sepsis progression and mortality, and allowed us to cluster differentiated phenotypes, offering potential for timely intervention and tailored therapeutic strategies (e.g., organ support for Group A, vasoactive agents for Group B).

A recent study by Boussina et al. [5] evaluated the COMPOSER deep learning model’s effectiveness in early sepsis diagnosis, applied to 6217 patients across two emergency departments at the University of California, San Diego. The model achieved a 1.9% reduction in sepsis-related mortality and a 5% improvement in treatment protocol adherence, with reduced organ damage within 72 h of sepsis onset, as indicated by SOFA scores. These findings support AI’s potential to improve clinical outcomes, though the authors stress the need for further validation across different healthcare settings and larger patient populations.

A further study from 2024 [30] describes the development and validation of MLM (XGBoost) to predict hospital mortality in patients with sepsis. Twelve clinical parameters were selected via LASSO regression to construct seven classification models. XGBoost showed the best performance, with an area under the ROC curve of 0.94, outperforming traditional methods such as the SOFA score. Model interpretation was facilitated using SHAP (Shapley Additive Explanations), which identified the main predictors of mortality as age, aspartate aminotransferase (AST), invasive ventilatory treatment, blood urea nitrogen (BUN) and neutrophil-to-lymphocyte ratio (NLR). The results suggest that integrating inflammatory biomarkers with ML algorithms can significantly improve predictive ability compared to conventional approaches.

An interesting paper [31] explores the use of AI to predict the risk of death in children with sepsis admitted to pediatric intensive care units (PICUs). Six machine learning methods including Artificial Neural Networks (ANN), support vector machines (SVM) and decision trees were used and compared on a retrospective dataset of 516 children from two hospitals. ANN proved to be the most effective method, with an accuracy of 0.96 in the test set and an AUC of 0.962, outperforming the other algorithms. The ANN model used a TensorFlow framework, structured with three fully connected layers, optimized for binary prediction (survival or death). Twelve clinical and laboratory parameters were analyzed, including lactate, blood pressure and pediatric SOFA index. ANN, compared to other scoring models such as SOFA, proved to have greater predictive power, specificity (0.99) and sensitivity (0.50), making it particularly suitable for the management of pediatric sepsis. The study concludes that the use of AI in PICUs may improve the ability to identify children at high risk of mortality early, facilitating targeted clinical interventions, even if it still needs further studies and external validation.

### 2.4. Personalized Treatment of Sepsis

Sepsis treatment is often complicated by diverse phenotypes and variable patient responses. AI has shown potential to enhance therapeutic strategies by enabling personalized treatment tailored to each patient’s unique characteristics.

Bataille et al. [32] explored using ML to better predict fluid responsiveness in patients with severe sepsis or septic shock, where precise fluid management is critical. Traditionally, fluid responsiveness is assessed with passive leg raising (PLR), but this method has limitations in certain patients. To overcome these, the researchers applied AI to echocardiographic data from 100 ICU patients. The MLMs developed, including neural networks and advanced regressions, were more accurate than traditional methods in predicting whether patients would benefit from fluid administration, identifying complex physiological patterns otherwise undetectable. This approach could improve fluid management in septic patients, reducing complications from improper fluid administration and ultimately enhancing survival rates and quality of care.

A further study [33] analyzes the clinical factors associated with the rapid treatment of sepsis, using MLM to identify the most relevant characteristics. The retrospective analysis is based on clinical data of adult patients (≥18 years) admitted to emergency departments over a 10-year period. Two Gradient Boosting Machine (GBM) models were developed for subgroups defined by the presence or absence of hypotension or elevated lactate (≥4 mmol/L). The models achieved high predictive performance with AUROCs of 0.91 and 0.84. Here, 760 variables were considered, including demographic data, vital signs, laboratory tests, comorbidities and diagnoses present at admission. In non-hypothesis patients, initial physiological parameters were more relevant, whereas in patients with fluid bolus requirements, extreme values (minimum and maximum) prevailed. The most impactful common characteristics included heart rate, temperature and blood pressure. The results suggest that ML can support clinical decisions, improving the speed of sepsis treatment by adaptive tools in EMRs.

Jie Yang et al. [34] detailed AI’s role in personalized sepsis treatment, particularly through reinforcement learning models. These models analyze previous therapeutic decisions and suggest optimal treatments based on patient-specific data, such as fluid and vasopressor administration—key interventions in sepsis management. AI has been shown to reduce the risk of fluid overload, a common sepsis complication, by enhancing clinical decision accuracy and better predicting patients’ responses to resuscitation. Real-time treatment response assessments further allow clinicians to adapt care promptly, improving therapeutic outcomes. This approach supports precision medicine by identifying patient subgroups that may benefit from tailored resuscitation strategies, especially for those with complex clinical manifestations where standardized treatments may be inadequate.

Ates et al. [35] recently presented a pioneering application of MLMs in personalized treatment of sepsis in the field of antibiotic therapy. They proposed MLMs as data-driven methods to find the connections between therapy effectiveness and patient data, managing to distinguish between healthy and sick states. By using this method, significant differences were observed between 48 and 72 h after dose adjustments (day 3–4). Cumulative analysis showed TDM led to lower mortality compared to the fixed-dose group, particularly within the critical first 72 h of sepsis management. Patients in the TDM group also experienced better recovery trajectories over 10 days, and the median SOFA scores upon ICU discharge were lower for TDM patients, suggesting less organ dysfunction. The method identified two recovery patterns: short-term benefits, with faster ICU discharge for a subset of TDM patients; long-term benefits, undergoing enhanced recovery rates and lower mortality in patients needing extended care. The evolutionary algorithm selected features like blood pressure, body temperature, creatinine, lactate, and piperacillin levels as critical for tracking recovery. This provided, indirectly, to disease classification and severity stratification, as well as offering a continuous and data-driven ‘‘multidimensional SOFA score”, offering a more nuanced view of patient states than conventional scores.

### 2.5. Relation Between AI and Providers: Strenghts and Possible Biases

MLMs’ performance can be influenced by the clinicians who use them, representing both advantages and limitations. Gonçalves et al. [36] documented nurses’ experiences with Laura^®^, a data-mining robot for early sepsis detection. Laura alerts healthcare professionals when patients’ MEWS (Modified Early Warning Score) indicators change, allowing for timely intervention. The study’s results were promising, as Laura improved diagnostic accuracy and professional satisfaction. However, challenges included the need for rapid data recording, healthcare professionals’ training in ML model use, and addressing distrust in the model’s accuracy.

**Table 1:** Selected studies for the review, including authors, AI models used, study design and study purpose in chronological order.

Title	Author	Year	AI-Driven Models Used	Study Design and Purpose
Impact of a deep learning sepsis prediction model on quality of care and survival	Boussina et al. [5]	2024	Deep-learning model (COMPOSER)	Observational study Impact of AI prediction tool on real-world patient outcomes
A Machine Learning Algorithm to Predict Severe Sepsis and Septic Shock: Development, Implementation, and Impact on Clinical Practice	Giannini et al. [8]	2019	Random forest classifier (machine learning algorithm) trained, validated and compared with clinical team	Retrospective and observational study Predict severe sepsis and septic shock and evaluate the impact on clinical practice and patient outcomes
Evaluation of a machine learning algorithm for up to 48-h advance prediction of sepsis using six vital signs	Barton et al. [9]	2019	Gradient-enhanced trees implemented with XGBoost	Retrospective study with cross-validation Early prediction of sepsis onset for early intervention and optimization of clinical monitoring
An Explainable Artificial Intelligence Predictor for Early Detection of Sepsis	Yang et al. [10]	2020	XGBoost (Gradient Boosting Trees)	Retrospective observational study Early and real-time prediction of sepsis in the ICU with interpretability for clinical decision support
Early detection of sepsis utilizing deep learning on electronic health record event sequences.	Lauritsen et al. [11]	2020	CNN-LSTM (deep learning architecture)	Retrospective multicenter cohort study Early detection of sepsis (up to 24 h prior)
DeepAISE—An interpretable and recurrent neural survival model for early prediction of sepsis.	Shashikumar et al. [12]	2021	Recurrent neural survival model DeepAISE (Deep Artificial Intelligence Sepsis Expert)	Comparative study with other four models for early prediction of sepsis
Effect of a sepsis prediction algorithm on patient mortality, length of stay and readmission: a prospective multicenter clinical outcomes evaluation of real-world patient data from US hospitals.	Burdick et al. [13]	2020	Gradient-boosted trees (XGBoost)	Multicenter prospective observational study Predict severe sepsis onset, improve clinical outcomes (mortality, length of stay, and readmissions)
A Machine Learning Sepsis Prediction Algorithm for Intended Intensive Care Unit Use (NAVOY Sepsis): Proof-of-Concept Study	Persson et al. [14]	2021	NAVOY Sepsis, prediction MLM crafted by using convolutional neural networks	Develop a high-performance ML sepsis prediction model viable and sharable from other hospital ICUs
Development and validation of a sepsis risk index supporting early identification of ICU-acquired sepsis: an observational study	Pappada et al. [15]	2024	Multilayer perceptron (MLP) artificial neural networks with Levenberg–Marquardt algorithm	Retrospective study Early diagnosis and risk prediction of sepsis and septic shock
FDA-Authorized AI/ML Tool for Sepsis Prediction: Development and Validation	Bhargava et al. [16]		Random forest calibrated	Multicenter prospective observational study Predict sepsis within 24 h and stratify patients according to risk to optimize clinical decisions
Diagnostic and prognostic capabilities of a biomarker and EMR-based machine learning algorithm for sepsis	Taneja et al. [17]	2021	Random forest	Two-center, observational cohort study Diagnose sepsis, stratify severity, and predict outcomes (hospital stay, mortality, readmissions)
A Machine Learning Model for Accurate Prediction of Sepsis in ICU Patients	Wang D. et al. [18]	2021	Random forest	Randomized controlled trial Develop an AI algorithm that can predict sepsis early
Diagnostic performance of machine learning models using cell population data for the detection of sepsis: a comparative study.	Aguirre and Urrechaga [19]	2022	Naïve Bayes (NB), K-nearest neighbor (KNN), support vector machines (SVM), random forest (RF), multi-layer perceptron (MLP), extreme gradient boosting machine (XGBOOST)	Prospective observational study Compare MLMs with classic LR model for diagnosing suspected sepsis using data from blood tests
Predicting sepsis onset using a machine learned causal probabilistic network algorithm based on electronic health records data	Valik et al. [20]	2023	SepsisFinder, a casual probabilistic network model	Observational cohort study Compare SepsisFinder’s ability to recognize sepsis from EHR data before NEWS2 used by clinicians
Real-time machine learning-assisted sepsis alert enhances the timeliness of antibiotic administration and diagnostic accuracy in emergency department patients with sepsis: a cluster-randomized trial	Kijpaisalratana et al. [21]	2024	Random forest	Cluster-randomized trial Early diagnosis and improved timeliness of antibiotic treatment in patients with sepsis
Machine Learning Algorithms Identify Pathogen-Specific Biomarkers of Clinical and Metabolomic Characteristics in Septic Patients with Bacterial Infections	Zheng et al. [22]	2020	XGBoost combined with variance threshold, maximal information coefficient (MIC) and relief	Retrospective observational study Develop an accurate diagnosis strategy among septic patients
Predicting Sepsis Mortality in a Population-Based National Database: Machine Learning Approach	Park YJ et al. [24]	2022	Logistic regression with Least Absolute Shrinkage and Selection Operator (LASSO) regularization, random forest (RF), gradient-boosted decision tree (Xg-boost), deep neural network (DNN), Super Learner (SL) model	Observational study Comparison between performance of conventional logistic regression approach and common MLMs in predicting sepsis mortality in retrospective data of patients diagnosed with sepsis and hospitalized
Supervised classification techniques for prediction of mortality in adult patients with sepsis	Rodríguez et al. [25]	2021	C4.5 Decision Tree, random forest, Artificial Neural Networks (ANN), support vector machine (SVM) models	Prospective multicenter cohort study Analyze and compare applicability and performance of various MLMs for classification and prediction of mortality in adult septic patients
Predicting in-hospital mortality in ICU patients with sepsis using gradient boosting decision tree	Li et al. [26]	2021	Gradient boosting decision tree (GBDT), logistic regression (LR), k-nearest neighbor (KNN), random forest (RF) and support vector machine (SVM)	Observational study Compare different MLMs to find the outperformer in selecting clinical and metabolomic features that distinguished septic patients from controls
Prospective, multi-site study of patient outcomes after implementation of the TREWS machine learning-based early warning system for sepsis	Adams, Henry et al. [27]	2022	Brand new ML-based clinical decision support tool for early prediction of sepsis, Targeted Real-Time Early Warning System (TREWS)	Prospective multicentric study Examine the association between patient outcomes and provider interaction with TREWS
Early Prediction of Mortality for Septic Patients Visiting Emergency Room Based on Explainable Machine Learning: A Real-World Multicenter Study	Park SW et al. [28]	2024	CatBoost, XGBoost and LightGBM	Multicenter prospective study with cross-validation Early mortality prediction in patients with sepsis to support clinical management and improve resource allocation
Interpretable machine-learning model for real-time, clustered risk factor analysis of sepsis and septic death in critical care	Jiang et al. [29]	2023	Logistic Regression Model (LRM) implemented with Python sklearn package, GBDT implemented using the XGBoost package	Retrospective observational cohort study Analyze sepsis and sepsis death risk factors to cluster differentiated phenotypes and offer tailored therapeutic strategies
Predicting sepsis in-hospital mortality with machine learning: a multi-center study using clinical and inflammatory biomarkers	Zhang et al. [30]	2024	XGBoost (eXtreme Gradient Boosting)	Multicenter retrospective study Predict hospital mortality in patients with sepsis and improve clinical decision-making
Establishment and Verification of an Artificial Intelligence Prediction Model for Children with Sepsis	Wang L et al. [31]	2024	Artificial Neural Network (ANN)	Multicenter retrospective study Predicting the risk of death in children with sepsis to support clinical decisions and improve prognosis
Machine learning methods to improve bedside fluid responsiveness prediction in severe sepsis or septic shock: an observational study.	Bataille et al. [32]	2021	Regression tree (CART), partial least-squares regression (PLS), neural network (NNET), and linear discriminant analysis (LDA)	Observational study Machine learning as a tool for predicting fluid responsiveness through transthoracic echocardiography (TTE) in critically ill patients
Clinical factors associated with rapid treatment of sepsis	Song et al. [33]	2021	Gradient Boosting Machine (GBM)	Retrospective observational study Identifying clinical factors influencing the rapid treatment of sepsis and improving clinical management through adaptive alerting tools in EMRs
Unraveling the impact of therapeutic drug monitoring via machine learning for patients with sepsis	Ates et al. [35]	2024	Different MLMs studied	Randomized controlled trial Propose a machine learning approach to measure the impact of therapeutic drug monitoring (TDM) on sepsis recovery
Factors driving provider adoption of the TREWS machine learning-based early warning system and its effects on sepsis treatment timing	Henry et al. [37]	2022	Brand new ML-based clinical decision support tool for early prediction of sepsis, Targeted Real-Time Early Warning System (TREWS)	Retrospective and observational study Analyze TREWS performance and impact of providers on the latter

A study by Henry et al. [37] examined how provider interaction could influence the TREWS model’s performance. Despite encouraging results, they observed that providers’ cognitive biases affected alert interactions. For example, clinicians often dismissed alerts for younger patients or during peak hours, while responding more promptly to alerts for older patients. Limitations included the retrospective design, limited alert criteria, and lack of generalizability. Future improvements should focus on enhancing providers’ familiarity and comfort with AI tools, to foster adoption in clinical workflows. This study illustrates how clinician-related factors can significantly bias the performance of well-designed models.

### 2.6. Safety

In the context of AI systems, reliability is primarily defined by the reduction of false positives, compliance with regulatory standards, and the mitigation of clinical risks such as alarm fatigue. It is evaluated using performance metrics such as the area under the ROC curve (AUROC), Mean Absolute Error (MAE), Root Mean Square Error (RMSE), F1 Score, Log Loss, or the ability to generalize to external datasets.

Among the models discussed, the Gradient Boosted Machine Learning Algorithm (GBMLA) for sepsis prediction seems to stand out for its high specificity, which reduces false positives and, consequently, alarm fatigue in hospital environments. Adherence to HIPAA standards for data de-identification ensures patient privacy protection, a critical safety consideration. However, the generalizability of models developed using Gradient Boost remains limited, as most results are derived from retrospective studies in academic settings. This raises concerns about their applicability in nonacademic or real-time clinical scenarios.

The Random Forest-Based Sepsis ImmunoScore model holds FDA approval, emphasizing its safety and efficacy. Its integration with electronic medical records (EMRs) reduces manual errors, enhances clinical safety and enables more accurate risk stratification. Nonetheless, external validation has been confined to specific US hospitals, limiting its generalizability to global healthcare contexts and increasing the risk of overfitting [16].

As previously mentioned, Giannini et al. [8] presented an algorithm with very high specificity (98%). However, its low sensitivity (26%) highlights a significant limitation—the potential to miss sepsis cases that could benefit from early intervention. This underscores the inherent challenge of balancing reduced alerts with the need to identify all critical cases. Another safety concern involves integrating the algorithm into clinical workflows—a key point emphasized in the study to prevent automation from negatively impacting human decision-making. The retrospective nature of validation and reliance on structured electronic health record data introduces uncertainty about its applicability in unstructured settings, or where data may be incomplete or inaccurate.

A recurring theme emerges from these examples: while MLMs show significant promise for improving patient safety and diagnostic accuracy, critical challenges remain. Chief among these is achieving a balance between sensitivity and specificity—essential to avoid missing sepsis diagnoses or generating excessive false positives.

Safety, however, is a multifaceted concept that extends beyond reducing false positives. It includes adaptability to real-world clinical environments without causing unintended consequences such as overtreatment or unnecessary antibiotic use. For instance, alert fatigue may diminish clinical staff responsiveness, while low sensitivity could delay crucial interventions.

Importantly, none of the studies reported any direct harm to patients during their use. The primary concerns revolve around data management, privacy protection, and workflow integration.

## 3. Discussion

In summary, AI and various MLMs have demonstrated significant potential in managing sepsis by enhancing the ability to predict, diagnose, and provide individualized treatment. MLMs have shown high accuracy in early prediction, with some algorithms capable of anticipating sepsis onset several hours in advance. Early detection offers clinicians crucial additional time for clinical decisions, potentially altering patient outcomes [38].

Two main approaches have emerged in sepsis prediction. The first leverages EHR data, which include longitudinal clinical and laboratory data, while the second relies on the continuous monitoring of vital parameters, such as heart rate, blood pressure, and oxygen saturation. These methods have complementary advantages: while EHR data provide a historical and contextual overview of a patient’s status, continuous monitoring allows rapid changes to be detected. However, few models integrate both sources; this approach could improve predictive accuracy even more.

From a diagnostic perspective, AI has demonstrated advantages over traditional scoring systems, such as SIRS, SOFA, and qSOFA, by identifying sepsis more quickly and accurately. In fact, with the ability to detect complex, nonlinear patterns, these algorithms achieve higher AUROCs, positioning themselves as promising tools for sepsis diagnosis and risk stratification.

Studies report an increased sensitivity and specificity of MLMs in diagnosing sepsis within settings like ICUs and EDs, as well as reductions in healthcare costs, hospital and ICU lengths of stay, complication rates, morbidity, and mortality—contributing to overall improved patient outcomes. The effectiveness of AI models also depends on the clinical setting. In ICUs, the main goal is to predict the outcome, and to optimize the treatment or determine its timing. On the other hand, in the EDs, sepsis prediction can guide the patient pathway, determining whether a high-risk patient should be transferred directly to the ICU or, in the case of lower risk, to less critical or sub-intensive wards. This means that the models should be not only center-specific, but also setting-specific.

Overall, AI presents substantial opportunities for personalizing sepsis treatment, enabling the timely initiation of antibiotic therapy and facilitating precise therapeutic management tailored to individual patient phenotypes. In all these scenarios, MLMs act not only as a prediction tool to predict at-risk patients, but also a pivotal decision-support tool to properly settle patient treatments and destinations [39].

Despite these advancements, the practical implementation of MLMs remains complex and poses critical challenges. Model performance for early sepsis prediction varies by clinical setting, generally showing the best results in environments with access to substantial, real-time clinical data, such as ICUs and EDs. This variability underscores that successful AI integration depends not only on algorithm quality, but also on the clinical setting and data accessibility. EHR data acquisition is typically straightforward, providing a foundation for effective model application across contexts. Key challenges include optimizing how MLMs utilize information, overcoming technical limitations in hospital IT integration, and accounting for costs, infrastructure, and necessary protocols.

Adoption among clinicians is another challenge, as many MLMs lack interpretability. The complexity of these models, often perceived as “black boxes”, can create reluctance among providers to incorporate them into daily practice. Some clinicians may be deterred by the risk of model error, which hinders successful implementation, though exceptions exist for particularly user-friendly models or those with high provider interest.

Elements like SHAP (Shapley Additive Explanation) values have been integrated to address this, helping to clarify the key variables influencing predictions. For instance, the DeepAISE model not only predicts sepsis risk, but also provides real-time analysis of risk factors, facilitating clinical adoption.

Behavioral obstacles also play a role. Studies, such as that by Henry et al., reveal that AI-generated alerts are often ignored during peak workload hours or in younger patients, highlighting the need for targeted educational initiatives to build confidence among healthcare providers.

A further issue is the low sensitivity and high false-positive rates in certain models. This imbalance between sensitivity and specificity poses significant clinical challenges; while false positives are minimized, the model may still fail to identify all sepsis cases, diminishing early prevention effectiveness. Schinkel et al. [40] highlights that false positives can lead to unnecessary treatments, antibiotic overuse, and antimicrobial resistance, straining healthcare resources. The authors emphasize the need for randomized clinical trials to validate these models’ effectiveness on a larger scale.

The transferability of these models to other healthcare settings or broader populations also requires rigorous external validation. A notable example is the study by Wong A. et al. [41], who attempted the external validation of the Epic Sepsis Model (ESM) to evaluate its impact on timely antibiotic administration. Unfortunately, their large-scale retrospective cohort study found that ESM had low sensitivity compared to existing clinical practice, causing significant alert fatigue without providing added clinical benefits. External validation is essential to assess a model’s reproducibility and generalizability prior to clinical adoption [39,42]. Nevertheless, it may not be possible to create a “universal” model due to center-specific variables.

We must be aware that each center may need to train its own model.

The integration of AI-based devices into clinical practice, particularly for complex conditions like sepsis, raises significant regulatory challenges. While AI holds immense promise, the regulatory frameworks governing its use are still evolving. In the US, the FDA categorizes AI-based devices as medical devices, requiring rigorous evaluations for safety, efficacy, and quality. This process includes multi-center validation, proof of improved clinical outcomes, and reliable operation across diverse settings. For example, the FDA-approved Sepsis ImmunoScore™ underwent extensive assessment, including analysis of large datasets and external validation. In Europe, the Medical Device Regulation (MDR) similarly requires evidence of clinical effectiveness and bias minimization. The proposed AI Act by the EU further stipulates strict criteria for transparency, safety, and efficacy, classifying AI systems based on risk levels. AI tools for sepsis, given their critical clinical applications, would likely fall into the “high-risk” category, necessitating rigorous validation and continuous audits. Global standardization remains a challenge, complicating efforts for companies to implement products across jurisdictions. Achieving uniform regulatory criteria would facilitate the broader adoption of AI technologies.

Beyond device regulation, data protection is crucial. AI models often require access to sensitive patient data, necessitating compliance with privacy regulations like GDPR in Europe and HIPAA in the US. Blockchain technology and tokenization, such as through Non-Fungible Tokens (NFTs), offer innovative solutions for ensuring data ownership and security. By leveraging blockchain, health information can be securely stored and accessed, ensuring patient trust and data integrity [7].

Additional challenges include the limited diversity of datasets for model training and the absence of standardized guidelines for model development, training, and validation in a healthcare setting. This lack of standardization risks inherent biases, which MLMs could inadvertently amplify.

Another notable limitation is the fact that some articles base sepsis definition on Sepsis-2, and some others on Sepsis-3; different studies apply varying clinical thresholds and diagnostic criteria for training, complicating the comparison of results. This lack of standardization in sepsis criteria hinders the evaluation of MLMs’ effectiveness and restricts their large-scale applicability.

In the end, in recent years, several companies have developed medical devices and software leveraging artificial intelligence (AI) to address the complexities of the early diagnsis and management of sepsis. Among them, Epic Systems has introduced the Epic Sepsis Model, one of the first AI algorithms implemented on a large scale. This tool utilizes electronic health record (EHR) data to identify patients at risk of sepsis. Despite its widespread adoption, independent validation studies have highlighted significant limitations, including relatively low sensitivity and a high rate of false positives. These issues have raised concerns about the model’s reliability and the potential for alert fatigue among clinicians [43].

Another prominent model is the Sepsis ImmunoScore™, developed by Prenosis Inc. and approved by the FDA. This innovative tool combines biomarkers, vital parameters, and laboratory data to calculate a personalized risk score, categorizing patients into risk levels ranging from low to very high. When untegrated into EHR systems, it enables the early identification of at-risk patients, significantly enhancing clinical management. Prenosis Inc. is recognized as an industry leader due to its focus on utilizing AI technologies for personalized diagnostic solutions [16].

A significant contribution comes from Sepsis Watch, a system developed by the Duke University Health System. Implemented in emergency departments, this deep learning model analyzes clinical data every 15 min, offering early warnings for patients at risk. Its integration has improved compliance with Surviving Sepsis Campaign guidelines, thus reducing mortality and enhancing the overall quality of care. Similarly, Dascena’s InSight system, also FDA-approved, stands out for its ability to predict sepsis up to 48 h before symptoms appear, showing substantial potential for prevention [44].

Another promising tool, COMPOSER, was developed at UC San Diego Health. This algorithm monitors over 150 clinical variables in real time to identify sepsis risk in emergency departments. Its implementation has been linked to significant reductions in mortality, exemplifying the value of AI in optimizing care in critical care settings [5].

Among approved AI solutions, the Targeted Real-Time Early Warning System (TREWS) stands out for its real-time clinical data analysis and machine learning algorithms, which help identify sepsis risks and support medical decisions. TREWS has demonstrated significant benefits, including reduced antibiotic administration time and improved survival rates, particularly in high-risk patients [27]. Although widely adopted, the Epic Sepsis Model’s performance appears inferior to advanced models like TREWS and COMPOSER.

Last but not leats, some models integrate clinical data and biomarkers with advanced algorithms, such as DeepAISE, which employs recurrent neural networks to provide accurate, interpretable predictions [12].

Overall, companies, academic institutions, and hospitals are collaborating to develop increasingly sophisticated tools. Despite these advancements, challenges related to standardization, validation, and large-scale integration still persist. Nonetheless, there is high interest in the potential of these technologies to revolutionize sepsis management.

## 4. Conclusions

AI’s safe and effective application in sepsis management could be realized through targeted improvements. Collaboration between AI systems and healthcare professionals could facilitate deeper data analysis, faster processing and optimized early decision-making. In clinical practice, the best results may be achieved by integrating MLMs as decision-support tools that complement, rather than replace, clinical judgment. Still, major limits to a deep comprehension of the algorithm’s output and the lack of generalization of the models must not be underestimated, making the topic of artificial intelligence ethics worth examining in depth.
